# Metabolic tumor volume and conformal radiotherapy based on prognostic PET/CT for treatment of nasopharyngeal carcinoma

**DOI:** 10.1097/MD.0000000000016327

**Published:** 2019-07-12

**Authors:** Zhaodong Fei, Chuanben Chen, Yingying Huang, Xiufang Qiu, Yi Li, Li Li, Taojun Chen

**Affiliations:** Department of Radiotherapy, Fujian Cancer Hospital, Fujian Medical University Cancer Hospital, Fuzhou, Fujian Province, People's Republic of China.

**Keywords:** biological target volume, F-18 fluorodeoxyglucose positron emission tomography/computed tomography, intensity-modulated radiation therapy, metabolic tumor volume, nasopharyngeal carcinoma

## Abstract

For patients with nasopharyngeal carcinoma (NPC), prognostic indicators to customize subsequent biologically conformal radiation therapy may be obtained via 2-(fluorine-18)-fluoro-2-deoxy-D-glucose (^18^F-FDG) positron emission tomography/computed tomography (PET/CT). This retrospective study assessed the prognostic significance and feasibility of conformal radiotherapy for NPC, based on ^18^F-FDG PET/CT. Eighty-two patients with NPC underwent ^18^F-FDG PET/CT prior to intensity-modulated radiation therapy (IMRT). The maximum standardized uptake value (SUV_max_) and metabolic tumor volume (MTV) of the primary tumor were measured, with MTV_*x*_ based on absolute SUV_*x*_ values ≥ specific threshold *x* on each axial image. The cut-off SUV_max_ and MTV values for predicting 3-year progression-free survival (PFS) were calculated according to a receiver operating characteristic curve. Assessed were correlations between SUV_max_ and MTV and between threshold *x* and MTV_*x*_, and the MTV percentage of the primary tumor volume at threshold *x*. The SUV_max_ and MTV were positively associated, as were MTV and primary tumor volume. Primary tumor volume, SUV_max_, and MTV were significant predictors of survival. The 3-year PFS rates for SUV_max_ ≤8.20 and >8.20 were 91.1% and 73.0%, respectively (*P* = .027). With furthermore analysis, patients having tumor with smaller MTV had higher 3-year PFS than patients having tumor with larger MTV. The 3-year PFS rate was inversely related to MTV. SUV_max_ and MTV, derived by PET/CT, are important for assessing prognosis and planning radiotherapy for patients with NPC. Small MTV indicated better 3-year PFS compared with large MTV. For the best therapeutic effect, MTV_4.0_ was the best subvolume to determine radiotherapy boost.

## 1 Introduction

Nasopharyngeal carcinoma (NPC) is a common malignant tumor of the head and neck that is especially prevalent in southern China, Malaysia, Thailand, and the Philippines.^[1,2]^ Radiotherapy is the primary treatment. For locally advanced NPC, the main reasons for failure of radical radiotherapy are distant metastasis and local recurrence.^[3]^

Oncologists have attempted to identify the subpopulation of patients with locally advanced NPC who may benefit from intensified treatment. Besides tumor-node-metastasis (TNM) stage, the primary tumor volume has significant prognostic value,^[4–7]^ with more aggressive therapy required for patients with larger tumor volume. Better local tumor control may be achieved by increasing the radiation dose to the tumor,^[8–10]^ but increasing exposure to the entire volume can cause serious complications, including nasopharyngeal necrosis and radiation-induced brain injury.

The functional and metabolic characteristics of specific regions of a malignant tumor may be obtained through medical imaging, via 2-(fluorine-18)-fluoro-2-deoxy-D-glucose (^18^F-FDG) positron emission tomography/computed tomography (PET/CT). Using ^18^F-FDG PET/CT, information regarding cell viability, proliferation, hypoxia, and apoptosis rate can be obtained.^[11]^ Local control of a tumor may be improved by boosting the radiation dose to selected portions of the primary target volume. The uptake of ^18^F-FDG in sub-volumes of the primary tumor can be monitored, and is valuable for diagnosis, staging, and early detection of NPC recurrence.^[12–14]^ For predicting prognosis, the standardized uptake value (SUV) and the metabolic tumor volume (MTV) are useful.^[15–18]^

This retrospective study assessed the prognostic significance of PET/CT parameters in patients with NPC who subsequently underwent intensity-modulated radiotherapy (IMRT). Also discussed is how ^18^F-FDG PET/CT may be utilized to increase the radiation dose selectively within a target volume to improve local control of NPC.

## 2 Methods

The Ethics Committee of Fujian Cancer Hospital granted approval of this retrospective study.

### 2.1 Patients

Eighty-two consecutive patients with newly diagnosed NPC were referred to our research center between October 2011 and December 2014 for whole-body ^18^F-FDG PET/CT (Table 1). The PET/CT scan was conducted within one week before treatment. Patients with any of the following were excluded from the present analysis: metastatic disease at diagnosis (M1 stage); other malignancies; prior treatment at other institutions; or in poor condition (Karnofsky index < 70%).

**Table 1:**
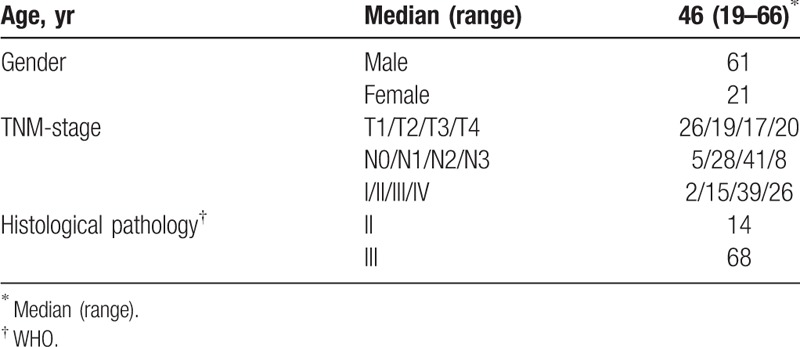
Patients’ characteristics, n.

The staging of each patient was determined via magnetic resonance imaging (MRI) and PET/CT findings, based on the seventh edition of the American Joint Committee on Cancer Staging Manual (2010).

### 2.2 ^18^F-FDG PET/CT imaging and parameters

The PET/CT scanning was accomplished with a Gemini TF 64 PET/CT scanner (Philips, Dutch). The ^18^F-FDG was manufactured by HM-10 cyclotron made by Japan Sumitomo Chemical. The purity of the radiochemical was >95%.

All patients fasted ≥6 hours before undergoing ^18^F-FDG PET/CT scanning. Serum blood glucose level was 3.9 to 6.5 mmol/L before ^18^F-FDG was intravenously administered at a dose of 148 to 296 MBq. Patients rested for 40 to 60 minutes in a dimly lit room before PET/CT scan. The CT scanning was from head to proximal thigh with the following acquisition parameters: 140 kV; 2.5 mA; matrix 512 × 512; and scan slice thickness 4 mm. The reconstructed PET images were obtained after applying the CT images for attenuation correction. Three images were then registered and viewed in a 3-dimensional (3D) model on an EBW 2.0 workstation.

The ^18^F-FDG SUV was based on the region of interest (ROI) of tumor lesions, and calculated as the decay-corrected tissue activity (nCi/mL) divided by the injected dose of FDG (nCi) and the patient's body weight (g). Primary tumors were delineated in 3-D mode using different SUV thresholds. That is, for the convenience of clinical practice, we determined the boundary of the volume of interest using SUV values ranging from 2.5 to 6.0, in 0.5 increments. The MTV of the primary tumor was calculated automatically under the various fixed thresholds of SUV, on MedEx software. For example, when the threshold of SUV was set at 2.5, the contouring margin comprised the entire primary MTV_2.5_, where the MTV was a quantitative measurement of ^18^F-FDG uptake based on the tumor lesions, the volume of interest. The MTV and SUV_max_ were acquired within the contouring margin around the tumor lesion.

### 2.3 Treatment

All the patients were treated with IMRT. Patients in stage I received radiation therapy only. Patients at stages II-IV B were administered 2 cycles of concurrent platinum-based chemotherapy (80 mg/m^2^, on day 1, every 3 weeks). Patients at stages III-IV underwent 2 cycles of neoadjuvant chemotherapy prior to radiotherapy. The neoadjuvant chemotherapy regimen consisted of gemcitabine (1000 mg/m^2^, on days 1 and 8) plus cisplatin (80 mg/m^2^, on day 2); or paclitaxel (135 mg/m^2^, day 1) plus cisplatin (80 mg/m^2^, day 2). Chemotherapy, including concurrent chemotherapy and neoadjuvant chemotherapy, were repeated every 21 days. Radiotherapy was performed simultaneously with the first cycle of concurrent chemotherapy. If necessary, the dose was modified according to interim toxic effects and the nadir blood counts during the preceding cycle. If the platelet count decreased to ≤25,000/mL or the leukocyte count decreased to ≤1000/mL, the doses of drugs were reduced by 25% in the subsequent cycle.

For IMRT, the target volumes were delineated using an institutional treatment protocol^[7]^ as follows. The primary gross tumor volume, and the involved lymph nodes, included all gross disease as determined by imaging, clinical, and endoscopic findings. The primary gross tumor volumes, and involved lymph nodes of the gross tumor volumes, were exposed to 69.7 Gy in 34 fractions at 2.05 Gy/fraction, in total. The clinical target volumes (CTV-1, CTV-2) were tissues that were considered to harbor the risk of microscopic disease. CTV-1 was exposed to 61.2 Gy at 1.8 Gy/fraction; CTV-2 and the involved lymph nodes of the CTV were exposed to 54.4 Gy at 1.6 Gy/fraction.

### 2.4 Follow-up

Follow-ups of the patients were conducted every 3 months during the first 2 years, every 6 months in the third to fifth year, and annually thereafter. All follow-ups included medical history, physical examination, and fiberoptic nasopharyngoscopy. Patients underwent head and neck MRI every 3 months in the first year, every 6 months in the second to fifth year, and annually thereafter. To detect distant metastasis of NPC, chest CT and abdominal ultrasonography were performed every 3 months during the first 2 years, every 6 months in the following year, and bone scintigraphy annually.

### 2.5 Statistical analysis

Statistical analyses were performed using SPSS 19.0 statistical software. The primary tumor volume and MTV were compared with the paired *t* test. A repeated-measures analysis of variance was used to analyze the different SUV thresholds for MTV. Receiver operating characteristic (ROC) curve analysis was used to determine the optimal SUV_max_ and MTV cut-off values. The optimal cut-off value was chosen as the best trade-off between sensitivity and specificity. Line correlation analysis was applied to reveal associations among the indices. The log-rank test was used to perform the univariate analysis, while the multivariate analysis was performed using the Cox proportional hazards regression model.

## 3 Results

### 3.1 Treatment outcomes

The median follow-up time was 36 months (range, 7–56 months). The locoregional and distant failure-free rates at 3 years were 91.5% and 92.7%, respectively. The 3-year rates of progression-free and overall survival were 82.9% and 91.5%.

### 3.2 Primary tumor volume

The primary tumor volume of NPC, equated with the gross tumor volume when strategizing a radiotherapy plan, was manually delineated slice-by-slice on the pretreatment contrast-enhanced MR images. The sum of the tumor volumes of all the slices was automatically calculated by computer, and defined as the primary tumor volume. The mean primary tumor volume in the present study was 37.74 ± 35.95 mL (range of 4.10–213.0 mL).

### 3.3 SUV_max_ and MTV

The average SUV_max_ was 8.25 ± 3.43 (range, 2.85–20.89). The MTV was a quantitative measurement of ^18^F-FDG uptake based on the tumor lesions, the volume of interest. The MTV is defined as an absolute SUV value that is equal to or greater than a given threshold on each axial PET/CT image. For example, where 2.5 is the threshold, MTV_2.5_ is defined as the absolute SUV that is equal to or greater than SUV_2.5_ on each axial PET/CT image. For this study population, the mean MTV values were as follows: MTV_2.5_, 20.53 ± 20.89 mL (range, 0.19–88.51); MTV_3.0_, 15.62 ± 17.08 mL (0–71.8 mL); MTV_3.5_, 12.26 ± 14.46 mL (0–60.54 mL); MTV_4.0_, 9.73 ± 12.51 mL (0–53.0 mL); MTV_4.5_, 7.83 ± 10.89 mL (0–46.14 mL); MTV_5.0_, 6.35 ± 9.43 mL (0–40.96 mL); MTV_5.5_, 5.17 ± 8.10 mL (0–36.80 mL); and MTV_6.0_, 4.24 ± 7.06 mL (0–33.02 mL). Thus, the MTV value was inversely associated with the threshold (*P* = .000). SUV_max_ and MTV_2.5_ showed a positive correlation, *r* = 0.562.

### 3.4 Primary tumor volume and MTV

**Figure 1:**
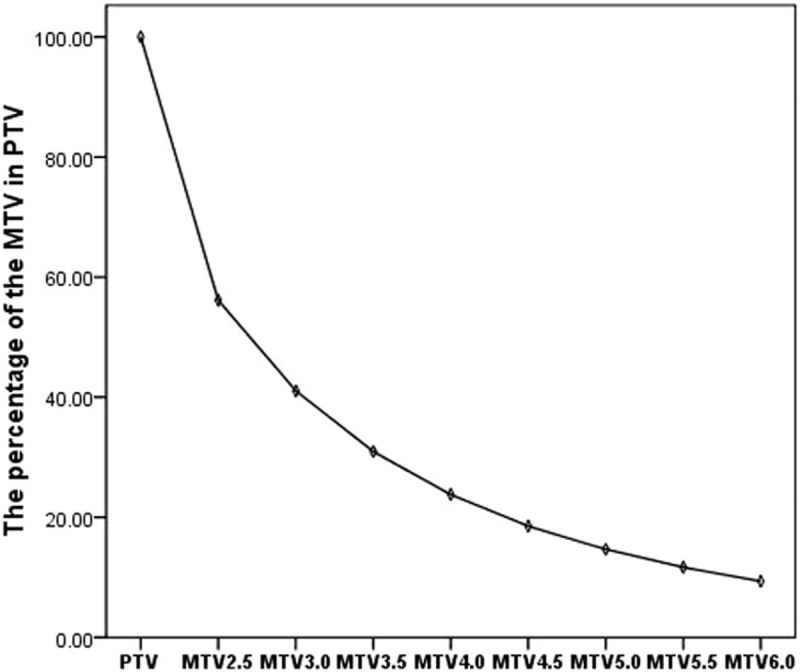
The percentage of primary tumor volume that constitutes the MTV. As the threshold increases, the area of the MTV within the PTV decreases. However, the change in MTVs above a threshold of 4.0 is not significant. MTV = metabolic tumor volume, PTV = primary tumor volume.

The MTV determined by PET is smaller than the primary tumor volume measured by MRI (*P* = .000). The primary tumor volume was positively associated with the MTV_2.5_ (*r* = 0.728). The percentages of MTV at each threshold in the primary tumor volume were as follows (Fig. 1): MTV_2.5_, 56.16 ± 30.05% (95% CI 49.55–62.76%); MTV_3.0_, 41.06 ± 25.92% (95% CI 35.37–46.76%); MTV_3.5_, 30.95 ± 22.96% (95% CI 25.91–36.0%); MTV_4.0_, 23.79 ± 20.26% (95% CI 19.34–28.24%); MTV_4.5_, 18.53 ± 17.90% (95% CI 14.59–22.46%); MTV_5.0_, 14.66 ± 15.75% (95% CI 11.20–18.13%); MTV_5.5_, 11.65 ± 13.68% (95% CI 8.64–14.65%); and MTV_6.0_, 9.33 ± 12.27% (95% CI 6.63–12.03%).

### 3.5 Three-year progression-free survival (PFS)

**Figure 2:**
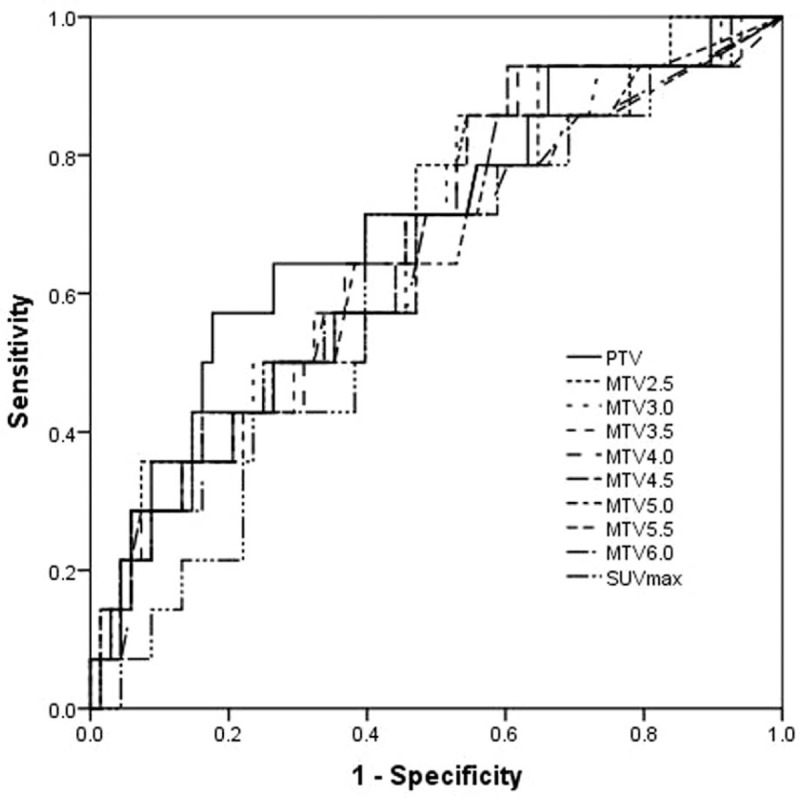
The ROC curve was used to determine the appropriate cut-off point of SUV_max_, PTV, and MTVs at thresholds 2.5–6.0. MTV = metabolic tumor volume, PTV = primary tumor volume, ROC = Receiver operating characteristic, SUV = standardized uptake value.

**Figure 3:**
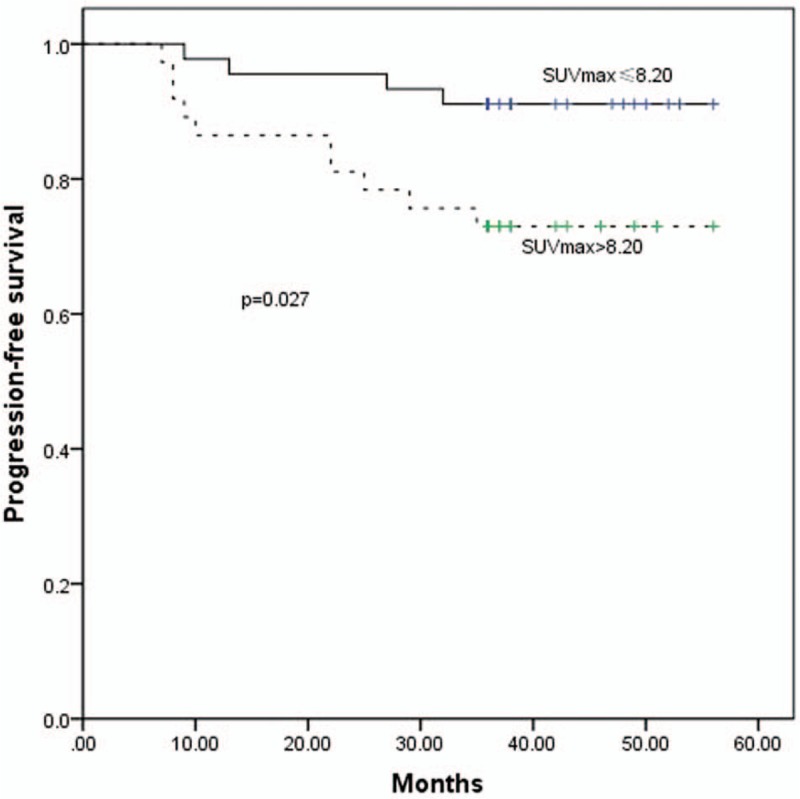
The PFS curves for SUV_max_ ≤8.20 and >8.20. PFS = progression-free survival, SUV = standardized uptake value.

**Figure 4:**
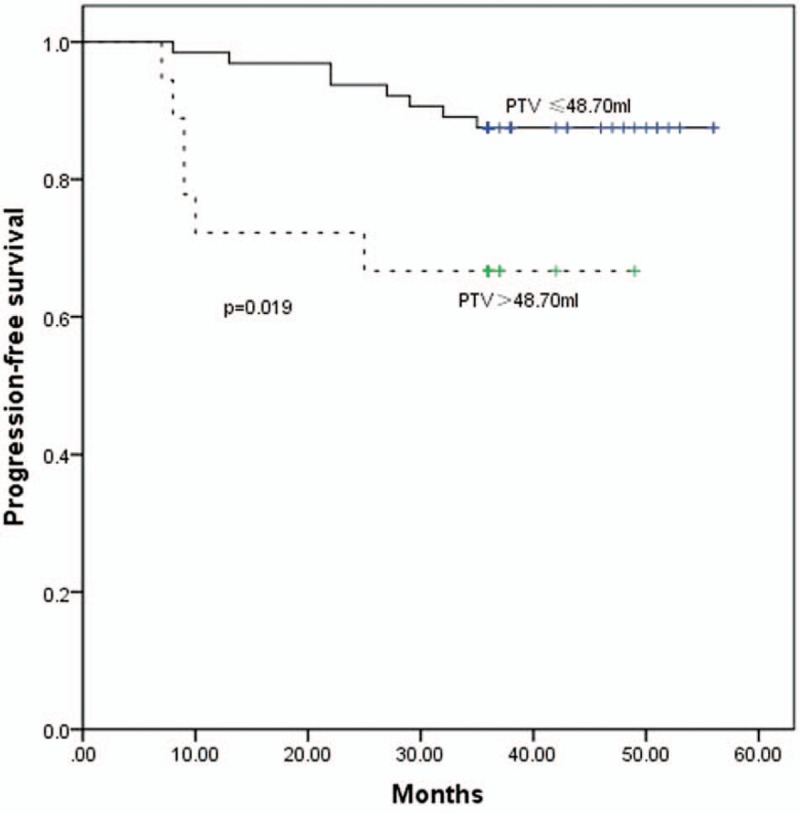
The PFS curves for PTV ≤48.70 mL and >48.70 mL measured by MRI. MRI = magnetic resonance imaging, PFS = progression-free survival, PTV = primary tumor volume.

ROC curves were calculated to determine the appropriate cut-off points for the SUV_max_, primary tumor volume, and MTV_2.5_-MTV_6.0_ for clinical application (Table 2; Fig. 2). The 3-year PFS rates for up to or greater than the cutoff point for SUV_max_ were, respectively, 91.1% and 73.0% (*P* = .027; Fig. 3); and for the primary tumor volume were 87.5% and 66.7% (*P* = .019; Fig. 4).

**Table 2:**
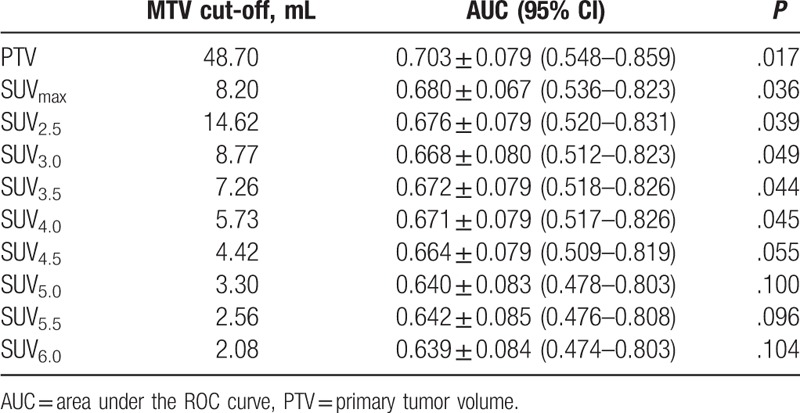
Cut-off points of the MTV.

**Figure 5:**
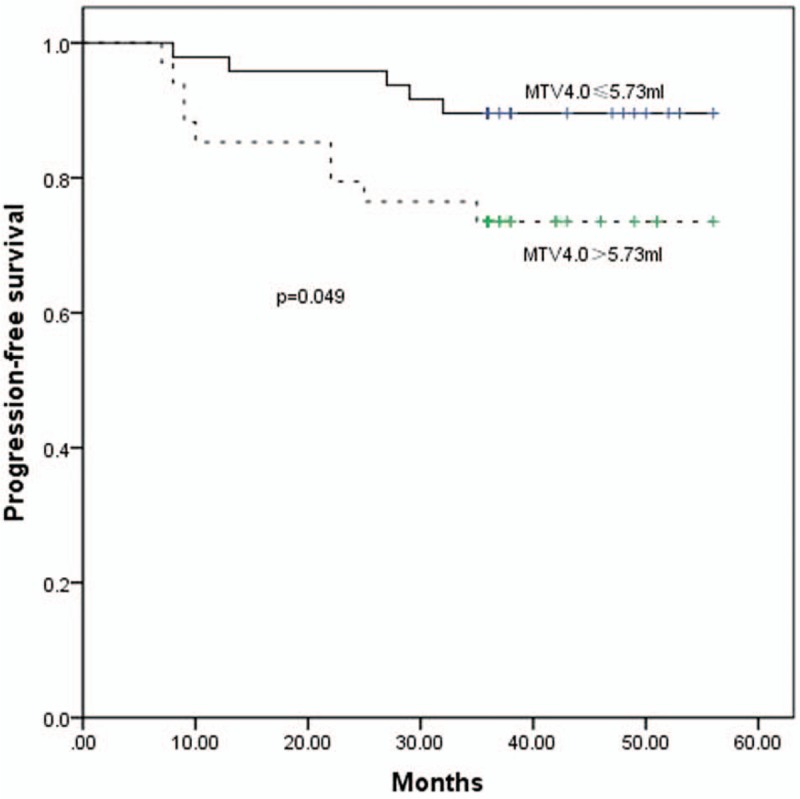
The PFS curves for MTV_4.0_ ≤5.73 mL and >5.73 mL. MTV = metabolic tumor volume, PFS = progression-free survival.

The 3-year PFS rates for up to or greater than the cutoff points for MTVs at thresholds 2.5–6.0 at 0.5 increments were the following: MTV_2.5_, 87.2% and 77.1% (*P* = .206); MTV_3.0_, 90.2% and 75.6% (*P* = .076); MTV_3.5_, 89.4% and 74.3% (*P* = .064); MTV_4.0_, 89.6% and 73.5% (*P* = .049) (Fig. 5); MTV_4.5_, 88.0% and 75.0% (*P* = .118); MTV_5.0_, 88.0% and 75.0% (*P* = .118); MTV_5.5_, 87.8% and 75.8% (*P* = .147); MTV_6.0_, 88.2% and 74.2% (*P* = .093).

### 3.6 Multivariate analyses

The multivariate analysis determined that the following were prognostic of poor PFS, where HR is the hazard ratio: primary tumor volume, HR = 3.022, *P* = .000 (95%CI 2.208–4.118); SUV_max_, HR = 1.811, *P* = .004 (95%CI 1.223–2.687); and N stage, HR = 1.762, *P* = .014 (95%CI 1.129–2.821). Neither MTV nor T-stage was a significant predictor for PFS.

## 4 Discussion

The objective of assessing prognostic risk factors is to guide clinicians to make a reasonable treatment plan. In NPC, TNM staging is the most important. However, recent studies have reported that TNM staging, as a unidimensional indicator, is limited by its inability to define the real, 3-dimensional tumor volume.

Many studies have suggested that the primary tumor volume,^[4–7]^ and PET/CT-derived parameters such as SUV and MTV, may have prognostic value in primary NPC.^[15–18]^ In particular, the SUV_max_ has been the most widely used, because of its reproducibility and low variability.

The present retrospective study applied ^18^F-FDG PET/CT data to confirm that the SUV_max_ of a primary NPC tumor is able to predict the outcome of treatment. The 3-year PFS rates for SUV_max_ ≤ 8.20 and >8.20 were 91.1% and 73.0%, respectively (*P* = .027). Lee et al. found that the best cut-off value of SUV_max_ to predict prognosis in NPC was 8.0, which is consistent with our result.^[19]^

The primary tumor volume is an excellent prognostic factor for NPC. The PET scan can comprehensively assess the tumor volume, and provides functional or metabolic information that cannot be obtained through MRI or CT imaging. One study reported that, compared with CT or MRI, the PET/CT image of the macroscopic surgical specimen was the most accurate for delineation of tumor volume in pharyngolaryngeal squamous cell carcinoma.^[20]^ The primary NPC tumor volume can be delineated, by using the functional and metabolic information gained from PET/CT. This should aid clinicians to estimate a prognosis and design a reasonable treatment plan.

SUV_max_ is a common and convenient parameter, but because it is a semiquantitative measurement of a single dot with the highest radiotracer concentration within the ROI, it does not reveal the heterogeneity within the tumor volume. The long-term follow-up analysis of the 82 patients in the present study showed that, at a fixed SUV threshold, the most discriminative MTV cut-off value had prognostic merit. Further analysis indicated that the 3-year PFS rate was inversely associated with the MTV. Other researchers have demonstrated similar results. Dibble et al confirmed that the MTV was a potential prognostic factor for predicting survival time in patients with oral and oropharyngeal squamous cell carcinomas.^[15]^ Chan et al found that the MTV was an independent risk factor in patients with metastatic NPC.^[18]^

In contrast to the SUV_max_, the MTV more accurately reflects the tumor burden. It is reasonable to suspect that the MTV can be used as the biological target volume, and the radiation dose should differ from that of the gross tumor volume. The functional information provided by this definition of the biological target volume should improve the therapeutic effect for patients with NPC who receive IMRT.

The threshold by which the MTV can be considered the biological target volume requires serious discussion. Yet, there have been few studies in this area—most studies have focused on the use PET to improve the target definition. A study by Liu et al of locoregionally advanced NPC showed that planning the IMRT dose based on FDG PET/CT guidance led to a significantly higher survival rate compared with using CT.^[21]^ In their study, they used SUV_50%max_ as the clinical standard for target volume delineation, and the basis for dose escalation. Wang et al used areas with SUV_2.5_ as the gross tumor volume; they concluded that PET/CT-guided dose escalation radiotherapy was tolerated well, and was superior to conventional chemoradiotherapy for locally advanced NPC.^[22]^ Both of these studies chose the SUV as the clinical standard for target volume delineation for dose escalation.

The MTV as measured by PET/CT is smaller than the gross tumor volume defined by MRI. Simultaneously, the MTV image provides functional and anatomical information, and indicates that portion of the gross tumor with the highest pace of revitalization.

Studies have shown that the major local failure pattern is central, that is, within the prescription-dose radiotherapy volume.^[23,24]^ Defining a functional sub-region of the gross tumor volume for radiotherapy boost is an attractive approach, which can improve results with no increase in radiotherapy toxicity. Lee et al reported that it was feasible, in 10 head-and-neck cancer patients, to escalate the dose to 84 Gy in hypoxic sub-volumes of the gross tumor without exceeding the normal tissue tolerance.^[25]^ A similar study of head-and-neck cancer from Hendrickson et al suggested that a higher dose to hypoxic sub-volumes significantly increased the probability of tumor control, without increasing the expected complications.^[26]^ However, in these studies the hypoxic sub-volumes of the gross tumor were subjectively determined and unquantified.

Our study revealed that, at any specific threshold, patients with a larger MTV experienced a poorer prognosis compared with those with a smaller MTV. This appears to theoretically support that the therapeutic effect may be improved if the gross tumor volume and MTV received separate radiation doses. Because of the small number of enrolled patients in our study, it was not possible to analyze more diverse MTV thresholds. However, we consider that the selected MTV should be a small percentage of the total gross tumor volume, to avoid necrosis induced by a high radiation dose. While the area of the MTV within the primary tumor volume decreased as the threshold increased, at thresholds >4.0, the change was not significant (Fig. 1). At the 4.0 threshold, the patients with a tumor with smaller MTV_4.0_ had a higher rate of 3-year PFS (89.6%) compared with patients with a larger MTV_4.0_ (73.5%). Thus, MTV_4.0_ may be most appropriate for defining hypoxic sub-volumes for boost planning. If the radiation doses to hypoxic sub-volumes are increased further, the SUV value corresponding with MTV should be increased simultaneously.

## 5 Conclusion

Our study indicated that SUV_max_ and MTV, derived by PET/CT, are very important in assessing prognosis and designing a radiotherapy plan in NPC. Furthermore, the 3-year PFS rate was significantly less for patients with SUV_max_ > 8.20. These patients require more aggressive treatment. Patients’ 3-year PFS rate was inversely associated with the MTV. Defining an appropriate MTV in the gross tumor volume for radiotherapy boost may improve the therapeutic effect, and MTV_4.0_ may be an appropriate definition for hypoxic sub-volumes for boost planning. The radiation doses to hypoxic sub-volumes, and the appropriate SUV value corresponding with the MTV, need to be investigated in further studies.

## Author contributions

**Conceptualization:** Zhaodong Fei, chuanben chen.

**Data curation:** Zhaodong Fei, chuanben chen, Xiufang Qiu.

**Formal analysis:** Zhaodong Fei, chuanben chen.

**Funding acquisition:** Zhaodong Fei, chuanben chen.

**Investigation:** Zhaodong Fei, chuanben chen.

**Methodology:** Zhaodong Fei, Yingying Huang.

**Project administration:** Zhaodong Fei.

**Software:** Zhaodong Fei, Yingying Huang, Xiufang Qiu, Yi Li, Li Li.

**Supervision:** Zhaodong Fei, chuanben chen.

**Writing – original draft:** Zhaodong Fei, chuanben chen, Yingying Huang, Xiufang Qiu, Yi Li, Li Li.

**Writing – review & editing:** Zhaodong Fei, chuanben chen, Yingying Huang, Xiufang Qiu, Yi Li, Li Li, Taojun Chen.

## References

[1] Torre LA Bray F Siegel RL Global cancer statistics, 2012. CA Cancer J Clin 2015;65:87–108. 25651787 10.3322/caac.21262

[2] Chua MLK Wee JTS Hui EP Nasopharyngeal carcinoma. Lancet (London, England) 2016;387:1012–24. 10.1016/S0140-6736(15)00055-0 26321262

[3] Lu H Peng L Yuan X Concurrent chemoradiotherapy in locally advanced nasopharyngeal carcinoma: a treatment paradigm also applicable to patients in Southeast Asia. Cancer Treat Rev 2009;35:345–53. 19211192 10.1016/j.ctrv.2009.01.002

[4] Mu-Kuan C Tony Hsiu-Hsi C Jen-Pei L Better prediction of prognosis for patients with nasopharyngeal carcinoma using primary tumor volume. Cancer 2010;100:2160–6. 10.1002/cncr.20210 15139059

[5] Lee CC Huang TT Lee MS Clinical application of tumor volume in advanced nasopharyngeal carcinoma to predict outcome. Radiat Oncol 2010;5:1–6. 20222940 10.1186/1748-717X-5-20 PMC2842277

[6] Guo R Sun Y Yu XL Is primary tumor volume still a prognostic factor in intensity modulated radiation therapy for nasopharyngeal carcinoma? Radiother Oncol 2012;104:294–9. 22998947 10.1016/j.radonc.2012.09.001

[7] Chuanben C Zhaodong F Jianji P Significance of primary tumor volume and T-stage on prognosis in nasopharyngeal carcinoma treated with intensity-modulated radiation therapy. Jpn J Clin Oncol 2011;41:537–42. 21242183 10.1093/jjco/hyq242

[8] Willner J Baier K Pfreundner L Tumor volume and local control in primary radiotherapy of nasopharyngeal carcinoma. Acta Oncol 1999;38:1025–30. 10665757 10.1080/028418699432301

[9] Bentzen SM Steepness of the clinical dose-control curve and variation in the in vitro radiosensitivity of head and neck squamous cell carcinoma. Int J Radia Biol Relat Stud Phys Chem Med 1992;61:417–23. 10.1080/09553009214551111 1347075

[10] Popple RA Ove R Shen S Tumor control probability for selective boosting of hypoxic subvolumes, including the effect of reoxygenation. Int J Radiat Oncol Biol Phys 2002;54:921–7. 12377346 10.1016/s0360-3016(02)03007-9

[11] Allal AS Slosman DO Kebdani T Prediction of outcome in head-and-neck cancer patients using the standardized uptake value of 2-[f]fluoro-2-deoxy-d-glucose. Int J Radiat Oncol Biol Phys 2004;59:1295–300. 15275712 10.1016/j.ijrobp.2003.12.039

[12] Kim G Kim YS Han EJ FDG-PET/CT as prognostic factor and surveillance tool for postoperative radiation recurrence in locally advanced head and neck cancer. Radiat Oncol J 2011;29:243–51. 22984677 10.3857/roj.2011.29.4.243 PMC3429909

[13] Minn H Clavo AC Grénman R In vitro comparison of cell proliferation kinetics and uptake of tritiated fluorodeoxyglucose and L-methionine in squamous-cell carcinoma of the head and neck. J Nucl Med 1995;36:252–8. 7830126

[14] Ou X Yang Z Hu C Use of 18F-FDG PET/CT in the diagnosis, staging, response assessment and prognosis of nasopharyngeal carcinoma: an updated review. Clin Cancer Res 2014;13:2627–33.

[15] Dibble EH Alvarez ACL Truong MT 18F-FDG metabolic tumor volume and total glycolytic activity of oral cavity and oropharyngeal squamous cell cancer: adding value to clinical staging. J Nucl Med 2012;53:709–15. 22492732 10.2967/jnumed.111.099531

[16] Higgins KA Hoang JK Roach MC Analysis of pretreatment FDG-PET SUV parameters in head-and-neck cancer: tumor SUV mean has superior prognostic value. Int J Radiat Oncol Biol Phys 2012;82:548–53. 21277108 10.1016/j.ijrobp.2010.11.050

[17] Schwartz DL Rajendran J Yueh B FDG-PET prediction of head and neck squamous cell cancer outcomes. Arch Otolaryngol Head Neck Surg 2004;130:1361–7. 15611393 10.1001/archotol.130.12.1361

[18] Sheng-Chieh C Cheng-Lung H Tzu-Chen Y The role of 18F-FDG PET/CT metabolic tumour volume in predicting survival in patients with metastatic nasopharyngeal carcinoma. Oral Oncol 2013;49:71–8. 22959277 10.1016/j.oraloncology.2012.07.016

[19] Lee SW Nam SY Im KC Prediction of prognosis using standardized uptake value of 2-[(18)F] fluoro-2-deoxy-d-glucose positron emission tomography for nasopharyngeal carcinomas. Radiother Oncol 2008;87:211–6. 18237806 10.1016/j.radonc.2008.01.009

[20] Jean-Fran?Ois D Thierry D Birgit W Tumor volume in pharyngolaryngeal squamous cell carcinoma: comparison at CT, MR imaging, and FDG PET and validation with surgical specimen. Radiology 2004;233:93–100. 15317953 10.1148/radiol.2331030660

[21] Liu F Xi XP Wang H PET/CT-guided dose-painting versus CT-based intensity modulated radiation therapy in locoregional advanced nasopharyngeal carcinoma. Radiat Oncol 2017;12:15. 28587681 10.1186/s13014-016-0739-y PMC5461636

[22] Wang J Zheng J Tang T A randomized pilot trial comparing position emission tomography (PET)-guided dose escalation radiotherapy to conventional radiotherapy in chemoradiotherapy treatment of locally advanced nasopharyngeal carcinoma. Plos One 2015;10:e0124018. 25915944 10.1371/journal.pone.0124018 PMC4411028

[23] Li JX Huang SM Jiang XH Local failure patterns for patients with nasopharyngeal carcinoma after intensity-modulated radiotherapy. Radiat Oncol 2014;9:87. 24674015 10.1186/1748-717X-9-87 PMC3986849

[24] Kong F Ying H Du C Patterns of local-regional failure after primary intensity modulated radiotherapy for nasopharyngeal carcinoma. Radiat Oncol 2014;9:60–160. 24552293 10.1186/1748-717X-9-60 PMC3936989

[25] Lee NY Mechalakos JG Nehmeh S Fluorine-18-labeled fluoromisonidazole positron emission and computed tomography-guided intensity-modulated radiotherapy for head and neck cancer: a feasibility study. Int J Radiat Oncol Biol Phys 2008;70:2–13. 17869020 10.1016/j.ijrobp.2007.06.039 PMC2888477

[26] Kristi H Mark P Wade S Hypoxia imaging with [F-18] FMISO-PET in head and neck cancer: potential for guiding intensity modulated radiation therapy in overcoming hypoxia-induced treatment resistance. Radiother Oncol 2011;101:369–75. 21872957 10.1016/j.radonc.2011.07.029 PMC3225491

